# Letter to the Editor

**Published:** 1911-09

**Authors:** H. L. Harris

**Affiliations:** Publicist, 100 William St., New York


### Letter to the Editor.

NEW YORK, August 15, 1911.

*To the Editor Medical Journal, Austin, Texas.*

SIR: I was interested in reading in your August issue an article copied from *The Lancet-Clinic* with the caption of "Endorsement of Dr. Wiley."

There seems to be an impression throughout the country that the resignation of Dr. Wiley from the Bureau of Chemistry would annul the pure food law. If such a supposition is correct, would not the same conditions prevail if Dr. Wiley should happen to pass away? No one can deny the fact that Dr. Wiley has been very active since the enactment of the pure food and drugs act. He has, in most cases, however, acted under instructions received from his superior, the Honorable Secretary of Agriculture, and if the public were as intimately acquainted with Dr. Wiley's qualifications as is the Secretary of Agriculture, it would clamor for Dr. Wiley's resignation. It is not generally known to the public that when Dr. Wiley was subpoenaed as an expert witness in the United States vs. Harper case, he was unable to qualify as a physician, a pharmacologist or a pathologist, and the officials in Washington were much chagrined when they learned this fact. It is a well known fact that Dr. Wiley's views on whisky, glucose, benzoic acid, etc., have been reversed by his superiors. These reversals would not have been made if Dr. Wiley's conclusions were considered scientific, neither would the referee board have been appointed if Dr. Wiley's investigations had been conducted in a scientific manner. The members of the so-called referee board are recognized as the foremost scientists in the United States, or one might say, in the world, and it is folly to criticize any conclusions arrived at by this board. Neither is it generally known that the Bureau of Chemistry is not mentioned in the pure food and drugs act except in one instance, where it says, "The Bureau of Chemistry shall make an examination of the samples and shall report its findings to the Secretary of Agriculture"; therefore the enforcement of the pure food and drugs act should be credited to Secretary Wilson.

The enforcement of the pure food and drugs act has certainly accomplished a vast amount of good in compelling manufacturers to correctly label drug and food products. It is estimated that 90 per cent of the judgments procured against various merchants were on account of mislabeling. In very few of the cases is there any evidence of food being adulterated with poisonous or injurious substances. Many of the States have practically copied the pure food and drugs act and State food commissioners have been and are quite active and vigilant in their endeavors to protect the consumer from adulterated food and drugs, all of which is without question excellent work.

The Bureau of Chemistry should not, however, be credited with all the good work. There is a bureau in the Department of Agriculture that has accomplished a vast amount of good, and about which little is heard. It is the Bureau of Animal Industry, the chief of which is Dr. A. D. Melvin. Dr. Melvin has been very active and has accomplished a great deal of good. In his annual report for 1910, "it is shown that nearly 1,000,000 animals were condemned in whole or in part, and in addition there were condemned on reinspection over 19,000,000 pounds of meat and meat products, which had become unwholesome since the inspection at time of slaughter.

There is really no danger to health and life from partaking of adulterated condiments, etc., and the bulk of food which mankind consumes, such as bread, meat, potatoes, fresh vegetables, fresh fruits, nuts, etc., never has been adulterated. Fresh meat, however, is a product that must be consumed before deterioration begins, as it is a well known fact that as soon as an animal is deprived of its life post-mortem changes take place, and unless meat is protected in some manner from the action of bacteria, it soon becomes infected with poisonous germs.

The law compels the truthful labeling of all foods and drugs when sold in packages, containers, etc., and the meat inspection law compels the proper stamping of all inspected meats.

There is also a vast amount of good being done in protecting the public from impure food in many of the cities.

The report of the Department of Health of New York City shows that during the past year 2,140,813 pounds of meat, fish, poultry and game were condemned. There was also condemned during the same period 20,370,841 pounds of fruit and vegetables.

The foregoing clearly demonstrates that the pure food law can be and is enforced in many instances without the assistance of Dr. Wiley, and if he is compelled to resign the pure food law will continue to be enforced, irrespective of statements to the contrary.

Yours very truly,

H. L. Harris,

Publicist, 100 William St., New York.

